# The hydrocarbon pollution crisis: Harnessing the earth hydrocarbon‐degrading microbiome

**DOI:** 10.1111/1751-7915.14526

**Published:** 2024-07-14

**Authors:** Robert Duran, Cristiana Cravo‐Laureau

**Affiliations:** ^1^ Universite de Pau et Des Pays de l'Adour, E2S UPPA, CNRS, IPREM Pau France

As part of the battle against climate change, the decarbonization of human activities has been acted in many countries worldwide. Thus, in order to limit the planet warming, it is expected to reduce the combustion of fossil fuels for decreasing drastically the production of greenhouse gas emissions. Although beneficial for reducing carbon dioxide (CO_2_) production to fight against climate change, this countermeasure unfortunately does not mean that hydrocarbon pollution is behind us because hydrocarbon pollution has many sources (Duran & Cravo‐Laureau, 2016) that will remain. It is estimated that the industrial and petroleum activities, which have already left behind a multitude of hydrocarbon‐contaminated sites that still need to be restored, release accidentally between 1.7 and 8.8 million tonnes of oil into the environment each year (Ambaye et al., 2022). The decarbonization is also expected to have a beneficial impact on decreasing the industrial release of hydrocarbons into the environment by reducing oil spill frequency and consequences (Little et al., 2021). In addition to the direct contribution of hydrocarbons resulting from the continued use of fossil fuels, the human activities also generate indirect inputs such as wildfires introducing polycyclic aromatic hydrocarbon (PAH) into the environment (Campos et al., 2019; Paul et al., 2023). Particularly “mega fires,” which burn large forest areas, are becoming more frequent as a consequence of climate change (Bracewell et al., 2023; van Oldenborgh et al., 2021). Of course, the natural sources of hydrocarbon contamination, such as volcanic activities and marine oil seeps, as well as biogenic sources (Duran & Cravo‐Laureau, 2016), continuously emit hydrocarbons into the environment. Thus, hydrocarbons last to be of concern for the environment in the future. In order to mitigate the impact of hydrocarbons on the environment, exploiting the hydrocarbon degradation potential that microorganisms have is a challenge to meet for scientists and engineers concerned about hydrocarbon pollution.

Important key knowledge has been gained on microbial hydrocarbon degradation as well as on the ecology of microbial communities inhabiting hydrocarbon‐contaminated sites. The hydrocarbon degradation capacity has been described for a large number of microorganisms from diverse terrestrial and aquatic environments. Several specialist hydrocarbon‐degrading microbial taxa have been described and isolated, as for example, the marine obligate hydrocarbonoclastic bacteria (OHCB) observed to bloom during marine oil spills (Yakimov et al., 2007), hydrocarbon‐tolerant fungi found in petroleum‐contaminated sediment (Álvarez‐Barragán et al., 2021), and alkane‐degrading specialist populations in soil (Hamamura et al., 2013). The characterization of specialist hydrocarbon‐degrading microbial taxa has unveiled the degradation pathways, both aerobic and anaerobic, for a wide range of hydrocarbon compounds from alkanes to PAHs (Chunyan et al., 2023). While gaining knowledge, various bioremediation strategies have been proposed to mitigate the impact of hydrocarbon in contaminated sites (Goñi‐Urriza et al., 2013). However, the different existing bioremediation processes for the in situ treatment of hydrocarbon‐contaminated sites have to content several technical, environmental, and regulatory hurdles before reaching optimal efficiency and societal acceptability (Boopathy, 2000). Particularly, the release of microorganisms, genetically modified or not, into the environment following a bio‐augmentation approach is controversial at both technical and ethical point of views (Lensch et al., 2024). Apart from the regulatory and ethical issues associated with the use of microorganisms for in situ bioremediation, which are not addressed here, there is no clear evidence that the added microorganisms colonize the contaminated site and effectively degrade the hydrocarbons (Wu et al., 2019). The difficulty for the added microorganisms to incorporate well‐established microbial community, known as colonization resistance in ecological theory, depends on several factors controlling the microbial community mixing, i.e., microbial community coalescence (Châtillon, Duran, et al., 2023; Rillig et al., 2015). These factors include the microbial communities legacy based on the history of contamination (Hafez et al., 2022), the size of the added community (Rillig et al., 2015), and priority effects (Tucker & Fukami, 2014). Nevertheless, the current knowledge on hydrocarbon microbial ecology provides crucial information opening the way for the implementation of bioremediation processes based on synthetic biology approaches (de Lorenzo, 2008).

The ecology of microbial communities living in hydrocarbon‐contaminated sites has been conceptualized on the basis of experimental and in situ observations. According to the disturbance theory (Allison & Martiny, 2008), the resistance, resilience, and functional redundancy of microbial communities in response to hydrocarbon contamination have been illustrated in several studies (Châtillon, Cébron, et al., 2023; Chronopoulou et al., 2013; Stauffert et al., 2014; Zerebecki et al., 2022). However, the microbial communities have been shown extremely dynamic and interactive in response to hydrocarbon contamination (Head et al., 2006; McGenity et al., 2012), requiring to switch beyond the concepts of resistance, resilience, and redundancy in order to better understand the structure or function relationships (Bissett et al., 2013). The ecological succession, microbial taxa replacement over time, has been observed in many cases after hydrocarbon contamination experimentally (Bordenave, Fourçans, et al., 2004; Bordenave, Jézéquel, et al., 2004; Cerqueda‐García et al., 2020) and in situ after an oil spill (Kimes et al., 2014; Péquin et al., 2022). Such ecological succession relies on the fact that hydrocarbon degradation is effected by a consortium of microorganisms, not all of which are directly involved in the degradation process. The microbial interactions have an extremely important ecological role in hydrocarbon degradation; hydrocarbon consumers receiving beneficial products (vitamins, EPS, metals, etc.) from associated microbes in return for their detoxifying activity (Louati et al., 2013; McGenity, 2014); even some associated microbes can also provide biosurfactants increasing hydrocarbon bioavailability that facilitates their degradation (Brito et al., 2009; McKew et al., 2007; Schweitzer et al., 2022). The microbial interactions also involve co‐metabolism and synergistic processes in which the degradation of hydrocarbons is enhanced by the use of a co‐substrate (Chen & Aitken, 1999; García‐Rivero & Peralta‐Pérez, 2008) or terminal electron acceptors (oxygen, nitrogen, sulphur) produced by other (micro) organisms (McGenity, 2014). Also, the microbial interactions allow microorganisms to colonize novel niches as illustrated by the inter‐kingdom interaction between fungi and bacteria (Álvarez‐Barragán et al., 2022), in which bacteria use the hyphosphere as ‘fungal highway’ for dispersal (Álvarez‐Barragán et al., 2023). Understanding the network of processes within microbial assemblages and the underlying mechanisms from which they arise is of paramount importance to achieve efficient bioremediation practices for oil‐polluted sites. It is accepted that trade‐offs drive microbial assemblages (Østman et al., 2014), particularly niche specialization has been recognized as key process in hydrocarbon degradation, each degradation step being performed by different microbial functional groups (Dalby et al., 2008). The establishment of ecotypes, step in niche specialization (Gushgari‐Doyle et al., 2022), has been demonstrated by the emergence of ecotypes adapted to either hydrocarbon structure (Kleindienst et al., 2015; McKew et al., 2007), temperature (Bargiela et al., 2015) or oxygen conditions (Terrisse et al., 2017). The genomic evolution conducting to niche specialization involves several adaptation mechanisms such as switch of integron gene cassettes (Abella et al., 2015; Huang et al., 2009) and horizontal gene transfer (Shahi et al., 2017); mechanisms that have been proposed to spread key degradation genes to harness microbial communities for hydrocarbon degradation in a synthetic biology approach (French et al., 2020).

In recent years, the concept of synthetic biology has been extended to microbial community (Borchert et al., 2021; De Roy et al., 2014) and even to the ecosystem (Hammond et al., 2023). The design of engineered microbial community, crucial step in the synthetic biology approaches, relies on microbial ecology principles and rules governing microbial assemblage processes such as community coalescence, habitat filtering, taxa replacement and turnover, and priority effects (Bernstein, 2019) for improving not only the biodegradation capacity but also favouring the colonization (Rocca et al., 2021; Ruan et al., 2024). Several strategies have been proposed to design synthetic microbial communities (SynComs) either by enrichment techniques resulting in simplified communities, defined as top‐down approaches, or reconstruction of microbial consortia by assembling bacterial strains, known as bottom‐up approaches (Bernstein, 2019; De Roy et al., 2014; Hu et al., 2022). Anent the top‐down approach, the current momentum for obtaining engineered microbiomes owning the desired functions can implement successive sub‐culturing under defined conditions (Li et al., 2023) applying as well high‐throughput techniques (Duran et al., 2022), or artificial selection applying sequential breeding (Swenson et al., 2000), or directed evolution subjecting community to perturbation cycles (Sánchez et al., 2021). The construction of SynComs, synthetic microbial assemblages, following a bottom‐up approach combines isolated microorganisms, genetically modified or not. The current efforts to build SynComs are based on the metabolic division of labour (MDOL) concept, the different steps of a metabolic pathway being performed by distinct populations in order to reduce the burden for each population (Tsoi et al., 2018). The design of SynComs also consider functional traits and potential niche occupancy (Jing et al., 2024), ecological coexistence (Chen et al., 2023), as well as microbial species interactions within the microbiome and outside the microbiome with organisms in its environment (Leggieri et al., 2021), that will benefit hydrocarbon bioremediation treatments. In order to direct the construction of microbial consortia, guide their transfer into the environment and anticipate their effects once released, SynComs draws largely on the recent developments of powerful computational genomic analyses (Jing et al., 2024), mathematical models (Tsoi et al., 2018), machine learning (Ghannam & Techtmann, 2021), and artificial intelligence (Patowary et al., 2023). The engineered microbial consortia have not only promising potential for developing technologies to reclaim hydrocarbon‐contaminated ecosystems (Yang et al., 2021), but they have also been proposed as tool for microbiome rescue to restore ecological stability in damaged ecosystems (de Lorenzo, 2018; Shade, 2023). Several stratagems and formulations have been proposed to deliver microorganisms and microbial consortia into contaminated sites to ensure their colonization and maintenance overcoming colonization resistance (Das & Chandran, 2011), which have been conceptualized as ‘Environmental Galenics’ (de Lorenzo, 2022). Most of them involve (bio)surfactants, foams, encapsulation, and immobilization of cells using supports such as biochar or nanoparticles (Mrozik & Piotrowska‐Seget, 2010; Patowary et al., 2023). Even, engineered horizontal gene transfer has been proposed especially for the treatment of large contaminated areas (de Lorenzo, 2022). Albeit the progress made in building artificial microbial communities, whether synthetic or not, to improve both degradation and colonization capacities for efficient bioremediation, some technical hurdles still need to be overcome to achieve effective bioremediation. Particularly, it is still required to improve the stability of microbial community by strengthening microbial interactions (Xu & Yu, 2021), as well as establish rules for a safe introduction of engineered microorganism into the environment (Wang & Zhang, 2019). The most glaring shortcoming is the lack of effective control of the artificial microbial communities in time, space, and composition for which promising strategies have been proposed relying, respectively, on signalling and adhesive systems, and symbiotic relationships (Grandel et al., 2021). Likewise, although it has been suggested that gene spread via horizontal transfer is self‐limited until fitness advantages over selection pressure persist (de Lorenzo, 2010), control systems should also consider the resilience of the local microbial community with respect to ecosystem biogeochemical cycling functions (French et al., 2020).

The evaluation of the performance of the bioremediation treatment requires robust monitoring methods to determine the efficiency of hydrocarbon degradation and removal, the effectiveness in reducing the toxicity, as well as the innocuousness of the applied technologies for human health and the environment. Thus, it is not enough to just assess hydrocarbon content in the contaminated environment by analytical chemistry methods but the monitoring toolbox must also include a battery of (eco)‐toxicity tests in order to appraise the ecological status (Schuijt et al., 2021). In the recent years, the development of microbial biomarkers for reporting the ecological status has gained momentum leading to the elaboration of microbial indexes such as microgAMBI (Aylagas et al., 2017), which adds the panoply of test. However, holistic approaches in line with the ‘One Health’ concept are recommended (Burke et al., 2017) for a comprehensive evaluation of the treatments' performances. In a world driven by economics, the implementation of bioremediation treatment for the rehabilitation of hydrocarbon‐contaminated sites, like any damaged site whatever the cause, must include collaboration with socio‐economic players within translational ecology framework (Enquist et al., 2017) and promote circular economy (Jagaba et al., 2022). The development of such initiatives will clearly demonstrate the financial benefits generated by taking care of environmental health, thus paving the way for the upturn of environmental engineering harnessing the Earth hydrocarbon‐degrading microbiome for reclaiming hydrocarbon‐contaminated sites.

## AUTHOR CONTRIBUTIONS

**Robert Duran:** Conceptualization; investigation; writing – original draft; writing – review and editing. **Cristiana Cravo‐Laureau:** Conceptualization; writing – review and editing.

## FUNDING INFORMATION

The research work of the authors was funded by the MAEWA project (H2020‐EU‐PRIMA programme, PRIMA‐P012‐0004‐01) supported by the French National Research Agency (ANR‐23‐P012‐0004‐01), the BIOMIC project (Interreg Sudoe Programme, European Regional Development Fund SOE4/P1/F0993), and the AQUASALT project (H2020‐EU‐ERANET‐MED programme, NMED‐0003‐01) supported by the French National Research Agency (ANR‐17‐NMED‐0003‐01).

## CONFLICT OF INTEREST STATEMENT

The authors declare no competing interests.
